# Aioli: Standardising Drugs in the FDA Adverse Event Reporting System (FAERS) to RxNorm and Anatomical Therapeutic Chemical (ATC) Codes

**DOI:** 10.1002/pds.70216

**Published:** 2025-09-04

**Authors:** Rowan E. Parry, Victor Pera, Katia M. C. Verhamme, Marcel de Wilde, Erik M. van Mulligen, Jan A. Kors

**Affiliations:** ^1^ Department of Medical Informatics Erasmus University Medical Center Rotterdam the Netherlands

**Keywords:** ATC classification, drug standardisation, FAERS, RxNorm

## Abstract

**Purpose:**

The Food and Drug Administration Adverse Event Reporting System (FAERS) is an important source of information on suspected adverse drug reactions, but does not standardise drugs. The Adverse Event Open Learning Through Universal Standardization (AEOLUS) System Provides Standardisation of drugs in FAERS to RxNorm, but its coverage leaves room for improvement and mapping accuracy has not been established. Furthermore, drugs are not mapped to the Anatomical Therapeutic Chemical (ATC) Classification System, which is frequently used in pharmacovigilance studies. Here we develop and evaluate the Aioli system, an extension of AEOLUS, to increase the mapping of drugs in FAERS to RxNorm, and to provide mappings to the ATC coding system.

**Methods:**

Several changes and extensions were made to the AEOLUS mapping process to increase the number of drugs standardized to RxNorm. Information in FAERS fields about ingredient, route, dose amount, dose form, and dose unit was used to map to the most appropriate ATC code. Mapping accuracy was assessed on an evaluation set of 122 FAERS records.

**Results:**

Aioli mapped 94.1% of drug names in FAERS to RxNorm, compared to 90.4% by AEOLUS. In addition, Aioli mapped 80.4% of drug names to ATC codes. Evaluation showed high accuracies, with 92.2% correct mappings to RxNorm and 94.0% to ATC.

**Conclusions:**

We have developed and evaluated the Aioli system that maps drugs in the FAERS database to RxNorm and ATC codes. With increased coverage of drugs and the mapping to ATC, Aioli further improves the usability of FAERS data in pharmacovigilance studies.

**Summary**

- The FDA Adverse Event Reporting System (FAERS) does not provide standardized drug names, complicating use of FAERS data in drug safety studies.
- The developed Aioli system maps drugs in FAERS to RxNorm and Anatomical Therapeutic Chemical (ATC) codes.
- Aioli maps 94.1% of drugs in FAERS to RxNorm (with 92.2% accuracy), and 80.4% to ATC codes (94.0% accuracy);
- With high coverage of drugs mapped to RxNorm and ATC, Aioli increases the usability of FAERS data.

## 1 Introduction

Spontaneous reporting systems are important sources of information on suspected adverse drug reactions (ADRs) [1, 2, 3, 4, 5]. These systems are used for signal detection and refinement in post‐marketing drug safety research and regulatory contexts [6, 7, 8]. One of the largest spontaneous reporting systems is the Food and Drug Administration (FDA) Adverse Event Reporting System (FAERS) [1]. FAERS is made publicly available and has been used in numerous post‐marketing surveillance studies.

Use of the data in FAERS has several issues, most importantly the presence of duplicate case reports and the lack of standardized drugs. Multiple data dashboards and tools have been developed to improve FAERS data access and quality, but often it is unclear if and how deduplication and standardization is done, or the software is not made available [9]. One of the few open‐source systems that does deduplication of data and standardization of drugs in FAERS is the Adverse Event Open Learning through Universal Standardization (AEOLUS) system [10]. AEOLUS standardizes drug names to the RxNorm coding system. Since RxNorm only covers drugs marketed in the United States (US), AEOLUS also uses an RxNorm Extension that includes drugs that are available in non‐US markets and was developed as part of the Observational Health Data Sciences and Informatics (OHDSI) project [11]. It was reported that AEOLUS is able to map 93% of the drugs mentioned in FAERS case reports [10], which leaves room for improvement. Also, the accuracy of the mappings was not yet investigated. Furthermore, no mappings are provided to the Anatomical Therapeutic Chemical (ATC) classification system, maintained by the World Health Organization (WHO) [12]. ATC is a frequently used coding system for drugs in observational health data studies, especially in Europe, and is commonly used in drug utilization and pharmacovigilance studies [13, 14].

In this study, we develop and evaluate an AEOLUS extension, called Aioli, to increase the mapping of drugs in FAERS to RxNorm, and to provide mappings to the ATC coding system.

## 2 Methods

**FIGURE 1:**
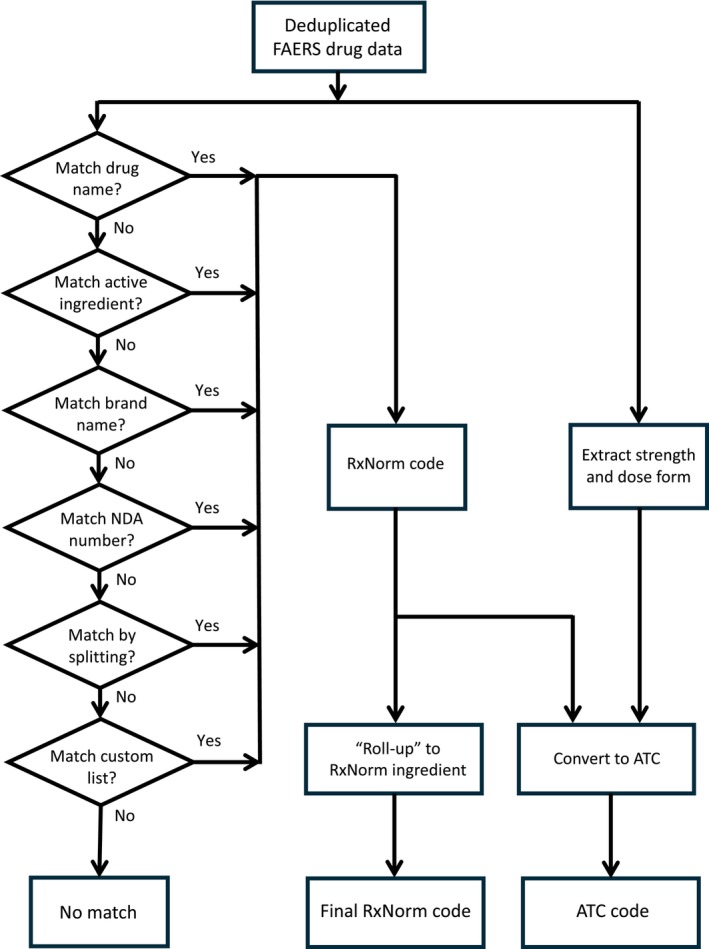
Flowchart of the mapping of drug records in FAERS by the Aioli system.

The Aioli system has been implemented as an extension of the AEOLUS system, replacing the AEOLUS mapping of drugs. The loading of FAERS data files, removal of duplicate reports, mapping of outcomes to Systematized Nomenclature of Medicine Clinical Terms (SNOMED CT) concepts, and generation of summary statistics about drug‐outcome relationships are still done in AEOLUS and have not been changed. Figure 1 shows the different processing steps in Aioli. In the following, we describe these steps, indicating the changes and extensions that were made to AEOLUS.

### 2.1 Mapping to RxNorm

The drug records in the FAERS reports are mapped to concept unique identifiers (CUIs) of standard code ingredients and clinical drug forms (for multi‐ingredient drugs) using the OHDSI Standardized Vocabularies version 5.3, which includes RxNorm and RxNorm Extension as the standard vocabularies for drugs [11]. For brevity, in the following, RxNorm will also imply RxNorm Extension. Aioli includes the mapping process that was defined in AEOLUS and consisted of a series of consecutive steps [10]. If a step resulted in a successful mapping, the mapping process for the given record was stopped; else, the next step was executed. The following steps were defined:

1. Using regular expressions, drug names (in field drugname in the FAERS data files) are matched against RxNorm preferred terms. The matching is performed iteratively, after subsequent removal of trailing spaces and dots, special characters (e.g., symbols, punctuation, parentheses), dosages, and routes of administration.
2. Active ingredients (in field prod_ai) are mapped to RxNorm CUIs.
3. Drug names are matched with brand names in the European Union Register of Medicinal Products. If a match is found, the corresponding active ingredient is mapped to an RxNorm CUI.
4. New Drug Application (NDA) numbers (in field nda_num) are linked to the active ingredient in the NDA Orange Book [15] and then mapped to RxNorm CUIs.
5. Drug names are split into separate words, and each word is matched with a multi‐ingredient, single‐ingredient, or brand name, and subsequently mapped to RxNorm.
6. Drug names are matched against a custom list of drug‐to‐RxNorm CUI mappings manually created with the OHDSI Usagi vocabulary mapping tool [16].
7. Matches in one of the previous steps may result in CUIs from a variety of RxNorm term types, such as ingredients, brand names, and dose forms. Using the relation table in the OHDSI vocabulary, each term type and CUI is converted (“rolled up”) to one of two final term types: single ingredient or multiple ingredients.

In Aioli, the AEOLUS mapping process described above was modified in several ways to increase the number of mapped drug names:

- The cleaning step was slightly expanded with additional characters to be removed.
- Matching was not only done against the preferred term for each RxNorm CUI, but also against all synonyms provided in the RxNorm rxnconso table.
- The European Union (EU) Register of Medicinal Products was replaced by the more comprehensive Article 57 database on all medicinal products authorized in the European Economic Area [17].
- The normalization function provided by the RxNorm Application Programming Interface (findRxcuiByString) was integrated to eliminate sources of discrepancies due to differences in word order, use of abbreviations, and American‐British spelling differences.
- A manually created custom list of 422 mappings from drug names to RxNorm CUIs was included.
- Minor fixes were made in the final roll‐up code to avoid incorrect mappings of brand names to clinical drug forms and of dose forms to drugs.
- A user option was provided to replace the final RxNorm code of a multi‐ingredient drug by the RxNorm codes of the single ingredients of that drug.

### 2.2 Mapping to ATC

ATC is a classification system in which active ingredients are hierarchically grouped by therapeutic use [12]. This means that a single active ingredient can have more than one code, depending on its therapeutic use, often related to drug strength or route of administration. For example, the drug finasteride in a low dose is used for treating male‐pattern baldness (D11AX10) and in a high dose for treating benign prostatic hypertrophy (G04CB01). As another example, the beta‐blocker timolol can be administered orally to treat cardiovascular conditions (C07AA06), while it is used in eye drops to treat glaucoma (S01ED01). For accurate mapping of FAERS drug records to ATC codes, it is therefore also necessary to consider the strength and route of administration of a drug.

The current approach to map drug names to RxNorm CUIs uses information from FAERS data fields about drug names, active ingredients, and NDA numbers. Mappings were made to any matching RxNorm term type. To map drugs to ATC codes, information in FAERS fields about route, dose amount, dose form, and dose unit is also used to derive four RxNorm components: ingredients, strength, dose form, and brand name. This information is then used to map to the most detailed RxNorm CUI possible, which is then converted to the most appropriate ATC code by means of an RxNorm‐ATC conversion table developed at Erasmus University Medical Center. If a drug has multiple ATC codes but the drug record contains insufficient information to determine a single ATC code, no ATC code is assigned.

### 2.3 Evaluation

Coverage is defined as the percentage of drug records for which a mapping was available. A unique drug record was defined by the unique combination of the following FAERS fields: drug name, active ingredient, route, dose amount, dose form, dose unit, NDA number. Missing values in these fields were treated as a separate, unique value. For example, if two drug records have the same values for all fields except for one field where one record has some specific value and the other record has a missing value, then the records are considered different. If these records would both have a missing value in that field, the records would be considered the same.

To assess the accuracy of the mapping process, we randomly selected 122 FAERS records. Two researchers (VP and KV) with a background in pharmacy, medicine, and drug safety research independently scored whether the Aioli mappings from FAERS to RxNorm and ATC were correct. Discrepancies between the observers were discussed and resolved in a consensus meeting. Accuracy is defined as the percentage of mappings that were correct according to the human observer. Interobserver agreement was determined by overall agreement, defined as the percentage of mappings on which the observers agreed, and by kappa, indicating the overall agreement corrected for chance agreement.

## 3 Results

We processed a download of the FAERS source data up to June 2023. After deduplication, there were 59,662,138 drug records across 16,485,294 case reports. Of these, 56,153,519 (94.1%) could be mapped to RxNorm by Aioli, whereas 53,909,090 (90.4%) were mapped by the original AEOLUS system. Aioli mapped 47,942,080 (80.4%) drug records to a single ATC code.

There were 4,227,336 unique drug records. Aioli mapped 3,394,928 (80.3%) of these unique drug records to RxNorm, and 2,776,885 (65.7%) to ATC.

Of the 122 drug records in the evaluation set, Aioli mapped 115 (94.3%) to RxNorm and 100 (82.0%) to ATC. There were various reasons why seven drug names could not be mapped to RxNorm, including a spelling mistake (cardiazem instead of cardizem), an orphan drug not marketed in the US and Europe (nimotuzumab), and several of the drug names with multiple ingredients where the mapping procedure was unable to find a matching multi‐ingredient RxNorm concept. Since RxNorm served as the basis for the consecutive mapping to ATC, these seven drug names were also not mapped to ATC. An additional 15 drug names could not be mapped to ATC, for example because the terms were too unspecific to assign an ATC code (e.g., calcium, potassium, triamcinolone), contained multiple ingredients in an unknown combination, or the ATC code was missing in the RxNorm–ATC conversion table.

The agreement between the two observers who evaluated the Aioli mappings to RxNorm and ATC was very good: 96.5% agreement and 0.73 kappa for RxNorm mappings, 95.0% agreement and 0.42 kappa for ATC mappings. After establishing a consensus reference standard, the mapping accuracy of Aioli was 92.2% (106/115) for RxNorm and 94.0% (94/100) for ATC. Of the nine RxNorm mapping errors, in five cases the mapped RxNorm string contained the correct ingredient but included dosage information that was not present in the original case record, in two cases the mapping was incomplete (e.g., isosorbide instead of isosorbide mononitrate), one case mapped to insulin regular whereas it should be insulin isophane, and one case contained conflicting brand name and ingredient information in the case record and was mapped to the wrong ingredient (omeprazole instead of pantoprazole). Of the six ATC mapping errors, three were due to an incorrect mapping to RxNorm. For the other three cases, an incorrect or related but less precise ATC code was assigned.

## 4 Discussion

The Aioli system described and evaluated in this study shows increased coverage of drug names in FAERS and provides accurate mapping of drug names to RxNorm and ATC codes. Aioli mapped 94.1% of drug names to RxNorm, compared to 90.4% by AEOLUS. In addition, Aioli mapped 80.4% of drug names to ATC codes, a capability that is absent in AEOLUS. The ability to map to ATC codes is particularly beneficial for pharmacovigilance studies in Europe, where ATC is a commonly used coding system. We have used this Aioli feature in several previous studies that analyzed FAERS data [18, 19].

Our evaluation of Aioli on a small subset of the mappings demonstrated high accuracies, with 92.2% correct mappings to RxNorm and 94.0% to ATC. These values can be compared with the interobserver agreement scores for RxNorm (96.5%) and ATC (95.0%), suggesting that there is still some room for improvement, in particular, for mapping drug names to RxNorm. Since ATC mapping is based on RxNorm, any improvement in RxNorm mapping will likely also benefit the mapping to ATC.

Our results for mapping drug names to RxNorm are difficult to compare with previous reports of tools for standardizing drug names in FAERS [9]. This is partly due to the steep increase in FAERS data over the years, which resulted in different FAERS data sets being analyzed in different studies. The effect of this can be illustrated by the coverage of drug names by AEOLUS, which was reported to be 93% in the original study (on about 5 million drug records) [10] but dropping to 90.4% in the current study (with almost 60 million drug records). Moreover, only very few studies have reported mapping accuracies. In an older study using the medication extraction tool MedEx on legacy data from the FAERS database [20], the mapping accuracy was 93.5% on a set of 200 drug names. In another study that used AEOLUS tooling [21], two annotators independently assessed 500 mappings and found accuracies of 96.6% and 96.8% for mapping to the correct active ingredient. In our study, we used a stricter annotation rule that penalized the inclusion of additional information not present in the case report. If only ingredient correctness were scored, 5 of the 9 RxNorm mapping errors of Aioli would be considered correct and the mapping accuracy would rise from 92.2% to 96.5%.

Our study has a number of strengths and limitations. A strength is that all of the code of the Aioli system is publicly available and can readily be applied by other investigators. Also, our evaluation of the mappings to RxNorm and ATC provides valuable information on the mapping accuracy and mistakes, while the interobserver agreement scores provide an indication of the maximum feasible accuracy that can be obtained by humans or the system. Finally, the mapping of FAERS data to ATC is new and facilitates the inclusion and analysis of FAERS data in pharmacovigilance studies that utilize the ATC coding system.

A limitation of our study is the relatively small evaluation set. A larger set would yield more reliable accuracy estimates and possibly offer more insight into the errors made by Aioli. Another limitation is that the RxNorm to ATC conversion table used in Aioli is only maintained by a single party (Erasmus University Medical Center). A more robust maintenance solution would be feasible if accurate RxNorm to ATC mappings were incorporated and maintained as part of the OHDSI vocabulary resources. Steps to accomplish this have recently been reported [22]. Such maintenance would include the mapping of new ATC codes, which are published annually, and adaptation to possible alterations in the ATC classification system, although the WHO is keeping these latter changes to a minimum [23]. Our mapping to RxNorm ingredients may also benefit from a large, manually curated drug name‐to‐ingredient dictionary that has recently been made available as a dynamic resource to foster knowledge sharing and collaboration among researchers developing drug name standardization tools [24].

In conclusion, we have developed and evaluated the Aioli system that maps drug records in the FAERS database to RxNorm and ATC codes. With increased coverage of drugs and the mapping to ATC, Aioli further improves the usability of FAERS data in pharmacovigilance studies.

### 4.1 Plain Language Summary

The Food and Drug Administration Adverse Event Reporting System (FAERS) is an important source of information on suspected adverse drug reactions, but does not use standardized drug terminology. The Adverse Event Open Learning through Universal Standardization (AEOLUS) system provides standardization of drugs in FAERS to RxNorm, but its coverage leaves room for improvement and its mapping accuracy has not yet been established. Furthermore, drugs in FAERS are not mapped to the Anatomical Therapeutic Chemical (ATC) classification system, which is frequently used in pharmacovigilance studies. Here we develop and evaluate the Aioli system, an extension of AEOLUS, to increase the mapping of drugs in FAERS to RxNorm, and to provide mappings to the ATC coding system. Aioli maps 94.1% of drugs in FAERS to RxNorm, compared to 90.4% by AEOLUS. In addition, Aioli maps 80.4% of drugs to ATC codes. On an evaluation set, Aioli shows high accuracies, with 92.2% correct mappings to RxNorm and 94.0% to ATC. With increased coverage of drugs and the mapping to ATC, Aioli further improves the usability of FAERS data in pharmacovigilance studies.

## Conflicts of Interest

The authors declare no conflicts of interest.

## Data Availability

All code of the Aioli system is publicly available with an Apache License 2.0 on https://github.com/mi‐erasmusmc/aioli.
